# Shifting Native Chemical Ligation into Reverse through N→S Acyl Transfer

**DOI:** 10.1002/ijch.201100084

**Published:** 2011-10-05

**Authors:** Derek Macmillan, Anna Adams, Bhavesh Premdjee

**Affiliations:** [a] Christopher Ingold Laboratories, Department of Chemistry, University College London20 Gordon Street, London WC1H 0AJ, UK phone: +44 (0)20 7679 4684 e-mail: d.macmillan@ucl.ac.uk

**Keywords:** acyl-transfer, native chemical ligation, peptides, protein modifications, thioester

## Abstract

Peptide thioester synthesis by N→S acyl transfer is being intensively explored by many research groups the world over. Reasons for this likely include the often straightforward method of precursor assembly using Fmoc-based chemistry and the fundamentally interesting acyl migration process. In this review we introduce recent advances in this exciting area and discuss, in more detail, our own efforts towards the synthesis of peptide thioesters through N→S acyl transfer in native peptide sequences. We have found that several peptide thioesters can be readily prepared and, what’s more, there appears to be ample opportunity for further development and discovery.

## 1. Introduction

The importance of peptides and proteins in biology and medicine has inspired chemists to consider methods that bring about their synthesis for over a century.[1] In most popular modern methods the peptide thioester, whether produced explicitly or transiently, plays a prominent role in the assembly of proteins and their analogues using convergent coupling strategies.[2] Native Chemical Ligation (NCL)[2b] has proved particularly useful, since it provides access to native proteins by coupling peptide thioesters with an additional component which usually, but not necessarily,[3] contains an N-terminal cysteine. Synthetic chemists have optimized efficient routes to peptide thioesters using *tert*-butyloxycarbonyl (Boc)-based methods of solid phase peptide synthesis (SPPS) and, until more recently, 9-fluorenylmethoxycarbonyl (Fmoc)-based methods lagged behind.[4] This is because thioesters themselves are not generally stable to the basic reaction conditions commonly employed for Fmoc removal and so a less direct path to thioesters is often required. Despite this obstacle, researchers have sought to develop efficient methods for thioester synthesis using Fmoc-based chemistry. The milder reaction conditions employed are generally considered more compatible with a variety of post-translational modifications and the additional, and sometimes chemically fragile, functionality embedded within various molecular probes.[4b]

**Scheme 1:**
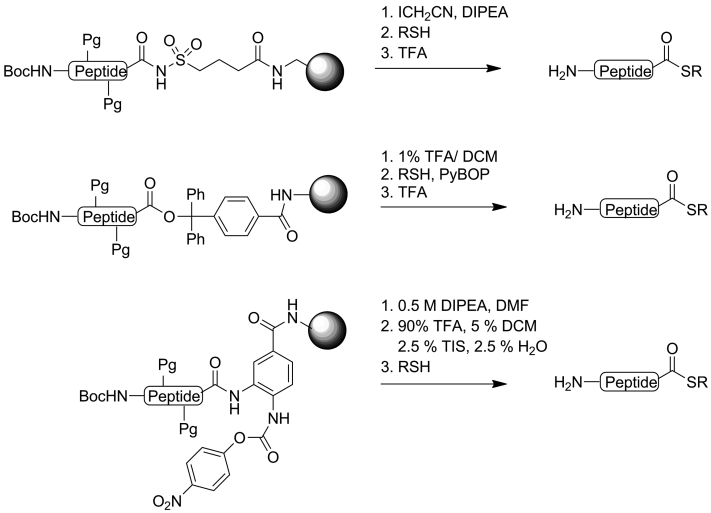
Some established and emerging Fmoc-based routes to thioesters. Pg=protecting group.

Using Fmoc-based SPPS, the thioester is usually isolated following chain assembly and activation of safety-catch resins,[5] formed after release of the otherwise fully protected peptides,[6] or formed from *N*-acyl urea terminated peptides[7] (Scheme 1). Each method appears to have its own advantages and disadvantages with respect to chemical yield, ease of monitoring, and user friendliness, but those described above currently appear the most reliable for the “routine” formation of long (>25 amino acid residues) peptide thioesters.

### 1.1. N→S Acyl Transfer

**Scheme 2:**
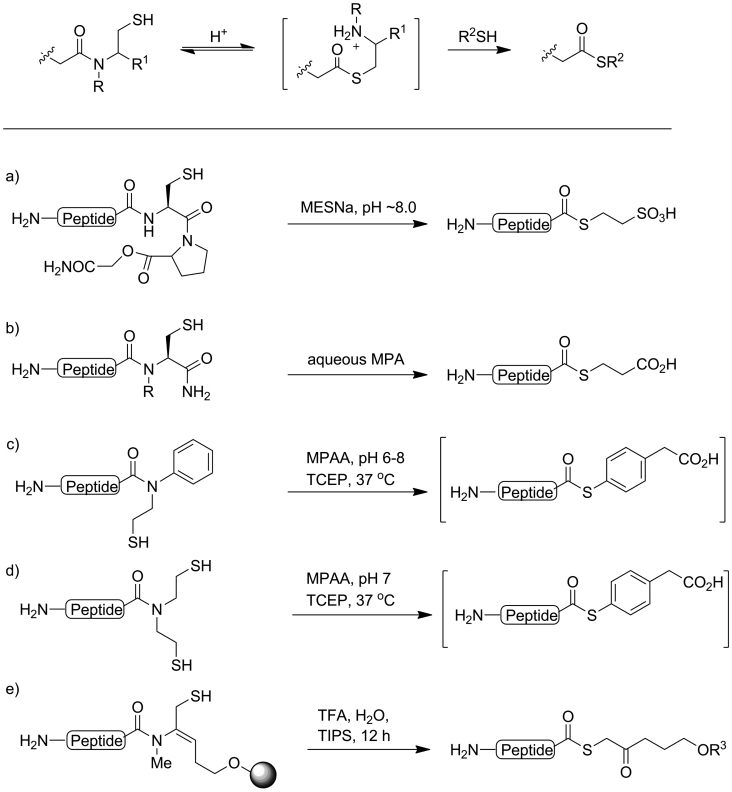
Schematic thioester formation through N→S acyl shift, and some new routes to peptide thioesters using a) the cysteinyl proline ester (CPE) method;[10] b) *N*-alkyl cysteine;[9h, i] c) *N*-sulfanylethylanilides (SEAlide);[9f, 11] d) *bis*(2-sulfanylethyl)amide peptides (BSEA);[9c–e] e) *N*-methyl enamides, R^3^=linker.[9g] Species depicted in square brackets are not isolated.

Recently, a number of methods that explore thioester formation via O→S[8] or N→S[9] acyl shift have been reported. Methods that involve N→S acyl shift seem particularly attractive since the nature of the acyl transfer-facilitating “device” can be simplified to cysteine and cysteine analogues or derivatives (Scheme 2). Furthermore, in some cases (Scheme 2 a, 2 c, and 2 d) a thioester precursor can be used directly in ligation experiments, producing the thioester only transiently as with *N*-acyl urea terminated peptides.

In most cases N→S acyl shift precursors contain a β-amino thiol motif. Since the equilibrium (depicted in Scheme 2) between the amide form of the peptide and the less stable thioester form (*S*-peptide) is expected to reside far to the left, this basic motif is “activated” by a number of attached groups. In each case the added group is believed to destabilize the scissile amide bond, making it more prone to thiolysis, and this is achieved in a number of ways. Generally:

- The scissile amide bond is N-alkylated (i.e., R=Me or Et). This influences the position of the N→S acyl shift equilibrium because the released secondary amine is more basic. Furthermore, the released secondary amine is less nucleophilic and so *S*-peptide formation is favored.
- The R-group can also allow additional delocalization of the amide nitrogen lone pair into an adjacent π-system, removing electron density from the amide linkage, resulting in a weaker amide bond.
- The R^1^-group has additional functionality that can intercept or transform the amino group that is released upon *S*-peptide formation, rendering the reaction irreversible.

Several of the developed acyl transfer facilitating devices include more than one of these features.

Derek Macmillan obtained his B.Sc. from the University of Edinburgh, then pursued a Ph.D. in bio-organic chemistry under the supervision of Professor Sabine Flitsch, also at Edinburgh. For postdoctoral studies (1999–2001) he joined the laboratory of Professor Carolyn Bertozzi at UC Berkeley. After returning to Edinburgh as research fellow he was awarded a Royal Society University Research Fellowship in 2003 and, following a short research visit to the laboratory of Professor Frances Arnold (CalTech), relocated to the Department of Chemistry at UCL in 2005. The Macmillan group explores the application of organic chemistry to understanding biological processes, with particular emphasis on the semisynthesis of modified proteins.

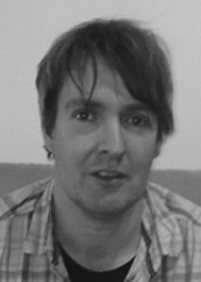

Anna Adams obtained her M.Chem. from the University of Southampton, UK (2005–2009). She subsequently was selected for the Wellcome Trust funded Ph.D. program at the Institute of Structural and Molecular Biology, Birkbeck College, University College London and the National Institute for Medical Research in London. For her doctoral degree she is working under the guidance of Dr. Derek Macmillan and in collaboration with Professor Surjit Kaila S. Srai, using chemical biology tools to study the peptide hormone hepcidin, in particular working on optimizing the selective formation of peptide thioesters for use in Native Chemical Ligation, whilst also seeking to develop biologically active analogues to hepcidin.

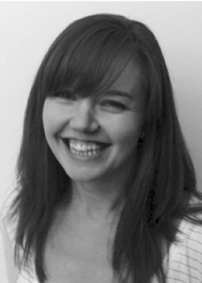

Bhavesh Premdjee completed his M.Sci. in Medicinal Chemistry at University College London (2006–10) and has since undertaken a Ph.D. under the supervision of Derek Macmillan. His research focuses on the synthesis of glycoproteins.

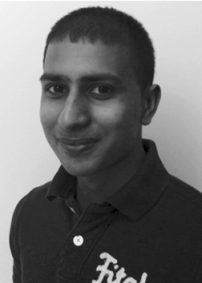

In our efforts to explore a scalable method for Fmoc-based SPPS of glycopeptide thioesters, we opted to first examine the cysteinylprolylester (CPE) method, reported by Kawakami and Aimoto.[10] We felt that this method was particularly attractive because the thioester was formed post-SPPS, allowing the precursors to be isolated in high yield and easily characterized. Additionally, since there was no requirement for specialized reagents or linkers, all aspects of the thioester production could be easily monitored.

**Scheme 3:**
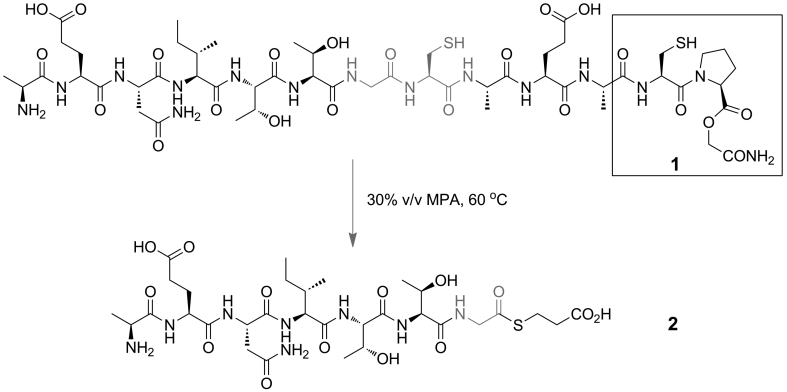
Exposure to CPE peptide **1** to 30 % v/v 3-mercaptopropionic acid at 60 °C for 48 h allowed the peptide thioester **2** to be isolated in 86 % yield.

We first prepared a short erythropoietin (EPO)-derived model peptide that was adorned with C-terminal CPE sequence (**1**, Scheme 3).[12] Upon exposure to typical reaction conditions (*N*-cysteinyl peptide, 2 % w/v MESNa, pH 7) we were disappointed to find that little reaction had occurred. Based on previous experience[13] and additional reports,[14] we predicted that the internal cysteine residue might, in some way, be influencing the process. When the peptide was resynthesized with the internal cysteine residue Acm protected, we found that the reaction was capable of proceeding as originally proposed, to form the Ala thioester. In order to “rescue” our non-Acm-protected peptide we decided to forcibly form the thioester by heating **1** to 60 °C in 30 % v/v 3-mercaptopropionic acid (MPA), as previously reported by Hojo[15] for mercaptomethyl prolyl esters. However, when the sample was heated we found that an alternative major product, **2**, emerged which corresponded to the Gly–MPA thioester, where the peptide had undergone thiolysis at the internal cysteine site. This result was initially surprising since we were unaware of any solely chemical method reported to bring about selective peptide or protein backbone cleavage with concomitant thioester formation.

**Scheme 4:**
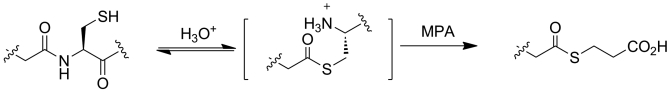
Thioester synthesis through N→S acyl transfer.

The fact that thiolysis appeared to have taken place selectively across the Gly–Cys junction (no smaller fragments could be observed) suggested that participation from the neighboring cysteine thiol, in a retro-NCL type process, was in operation (Scheme 4). Presumably any peptide or protein sample that contains cysteine could be a substrate, and indeed fully reduced erythropoietin (EPO), a 166-residue protein containing four cysteines (Cys7, Cys 29, Cys33, and Cys 161), was shown to undergo cleavage to afford thioesters. Surprisingly, only two thioesters could be observed, corresponding to residues [Ala1–Gly28]–COSCH_2_CH_2_CO_2_H, and [Ala1–His32]–COSCH_2_CH_2_CO_2_H. There was no evidence for cleavage at the Ile6–Cys7 junction or the Ala160–Cys161 junction, and this led us to predict that some selectivity might be achievable in thioester formation across Xaa–Cys junctions. When 66 KDa bovine serum albumin (BSA), containing over 30 cysteine residues, was subjected to similar MPA treatment, this protein underwent significant and complex fragmentation in only 1 h.

These observations led us to the realization that peptides simply equipped with a C-terminal cysteine should be substrates for thioester formation. The fact that the reaction occurs at all is presumably because the N→S acyl shift is facilitated by enhanced protonation of the liberated amino group below pH 7.[16] Further model reactions confirmed that thioester formation could be selective, particularly across Gly–Cys, His–Cys, and Cys–Cys junctions, and that in these cases the products do not appear to undergo epimerization of the C-terminal residue (Table 1).

**Table 1:** Peptides designed to test for selectivity in MPA-mediated fragmentation[12]

Peptide sequence	X^8^C cleavage		X^12^C cleavage		X^12^C : X^8^C	Isolated 11 mer yield %
				
	calc. m/z	obs. m/z	calc. m/z	obs. m/z		
H–AENITTGCAEHC–NH_2_	793.3	793.5	1233.5	1233.6	≍1:1	28
H–AENITTGC(Acm)AEHC–NH_2_	793.3	n/o	1304.5	1304.6	>9:1	39
H–AENITTICAEHC–NH_2_	849.4	n/o	1289.5	1289.7	>9:1	60
H–AENITTICAEGC–NH_2_	849.4	n/o	1209.5	1209.7	>9:1	n.d.
H–AENITTGCAEGC–NH_2_	793.3	793.5	1153.4	1153.6	≍1:1	26
H–AENITTGCAECC–NH_2_	793.3	793.5	1199.4	1199.6	≍1:1	28
H–AENITTGCAEIC–NH_2_	793.3	793.5	1209.5	n/o	1:9	n.d.

In substrates where the Xaa of an Xaa–Cys motif was a β-branched amino acid, there was no appreciable thioester formation. It is noteworthy that alternative Xaa–Cys motifs such as Ala–Cys, Ser–Cys, and Phe–Cys all appeared capable of forming thioesters under more forcing conditions, though the potential for epimerization of the terminal amino acid was not explored in detail. In one example, epimerization of the Ala residue appeared to have occurred to a significant extent (>10 %). The presumed epimeric product was confirmed by synthesis of the d-Ala-terminated peptide and subjecting it to identical reaction conditions. The retention time of the thioester derived from the d-Ala terminated peptide was identical to that of the epimeric product observed when using l-Ala. The d-Ala-derived thioester had also epimerized to a significant extent. No similar phenomenon was observed when using peptides terminating in His–Cys, or Cys–Cys.[17]

What also became apparent from these studies was that some species could not be easily observed by HPLC when using MPA as the reagent to effect thioester formation. MPA has a tendency to precipitate from the reaction mixture upon extended reaction time, and additional MPA-derived peaks are observed in the HPLC trace as the reaction progresses. Furthermore, the acidity of MPA appeared incompatible with Asp residues, as also reported by Hojo,[15] and so it became clear that new reagents and reaction conditions would need to be investigated.

## 2. N→S Acyl-Transfer in Native Peptide Sequences

### 2.1. Some Peptide Features That Affect N→S Acyl-Transfer in Native Peptide Sequences

So far, formation of thioesters via N→S acyl transfer at Cys-terminated peptides has been shown to proceed more efficiently when a peptide possesses a His, Gly, or Cys residue adjacent to the C-terminal Cys.[12] It is probably not a coincidence that synthetic peptide thioesters adorned with these residues at the C-terminus are also known to react fastest in NCL reactions.[18] When Xaa–Cys motifs occur within a peptide sequence, thioester formation can potentially occur more widely, but often with reduced efficiency. Consequently, while Ala–Cys-terminated peptides can undergo slow thioester formation under normal conditions (with accompanying epimerization), this reaction appears extremely inefficient when the Ala–Cys motif is situated internally.

An obvious limitation is that when additional especially labile sites exist within a peptide, these Cys residues must be protected to prevent unwanted cleavage, or the adjacent residue (e.g., Gly) must be substituted (e.g., with Ala). Although we have observed faster thioester-forming rates when the scissile site is at the C-terminus of a peptide, rather than situated internally, and prepared as a C-terminal carboxylic acid rather than a carboxamide,[17] this difference in rate is not sufficient to ensure that reaction occurs selectively at the C-terminus. As a consequence, it is a priority to discover new reagents, additives, reaction conditions, or C-terminal motifs, such that the process can be improved, although it would be ideal if the optimal Xaa–Cys motif could be genetically encoded such that biologically derived proteins could be processed.

Asp–Cys sequences were poorly tolerated when heated to 60 °C in MPA, and substrate peptides can undergo complete hydrolysis at the Asp residue over prolonged reaction times.[19] This undesirable reaction can be completely abolished if the conservative substitution from Asp to Glu is made, although this is not practical for large proteins. Forming thioesters in Asp-containing peptides at pH 5.8 is currently a compromise solution since performing the reaction at this pH reduces hydrolysis at Asp.

A further current limitation is that long peptides are often particularly prone to precipitation or aggregation upon periods of prolonged heating, so for the reaction to be widely applicable to recombinant samples, it is essential that reaction conditions are developed that allow thioester formation to proceed at lower temperatures. The reaction can be conducted in 6 M guanidine**⋅**HCl, but while the samples may remain in solution throughout the experiment, thioester formation can remain inefficient.

While there are clearly still significant obstacles to overcome, several short (10–15 residues) peptide thioesters can be routinely prepared, using regular peptide sequences as thioester precursors, in good yield. Using thioesters derived from this novel approach, we have already assembled biologically active samples of human β-defensin 3 (hBD3),[20] and the iron-regulatory peptide hepcidin.[21] The simplicity with which the precursors can be prepared has propelled us forward to investigate satisfactory solutions to the current limitations, since they do not appear overtly intractable.

### 2.2. Optimal Conditions and Reagents

**Figure 1:**
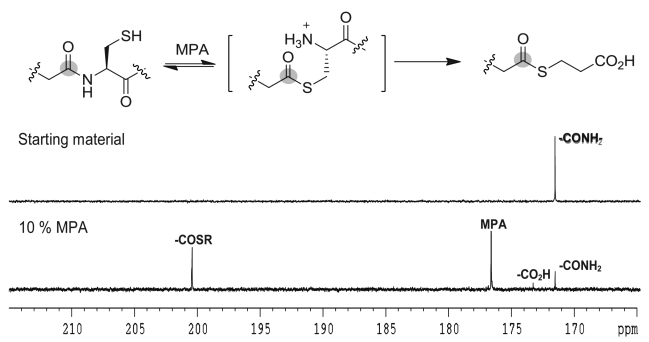
^13^C NMR analysis of a Gly ^13^C-1 labelled Gly–Cys motif undergoing thioester formation.[20]

In order to initiate optimization studies we needed a useful handle with which reaction progress under varying conditions could be compared. The previous work of Aimoto[22] and Danishefsky[8b] suggested that employing a ^13^C-1 labelled Gly–Cys motif would be useful, owing to the significant difference in ^13^C NMR chemical shift of the carbonyl carbon upon conversion from ^13^CONHR (approx. 170 ppm) to ^13^COSR (approx. 200 ppm). Usefully, hydrolysis (^13^CO_2_H) could also be observed (Figure 1).[20]

Interestingly, the ^13^C NMR spectrum appeared free of any intermediates, suggesting that N→S acyl transfer is rate-determining. However, although the *S-*peptide has not been isolated, it appeared essentially indistinguishable from the thioester product by ^13^C NMR, and it is unclear to what extent it accumulates during the reaction. A reaction that appears complete by ^13^C NMR can return significant quantities of starting material following HPLC, suggesting that the *S*-peptide exists in solution and reverts to amide upon purification.[22] These findings then suggest, that the transthioesterification, rather than the N→S acyl shift, can determine the rate of the reaction. Both steps would be affected by changes in pH, and the precise mechanistic details of the reaction require a more thorough investigation.

^13^C NMR spectroscopy, followed by LC-MS to confirm the product distribution, became a useful way to examine thioester formation under a varying reaction conditions.[20] Upon screening a small selection of water-soluble thiols, sodium 2-mercaptoethanesulfonate (MESNa) quickly emerged as the better thiol additive. Not only did it have good solubility in water and was free of the stench, it also caused less hydrolysis of the thioester product than MPA. Moreover, unlike MPA, MESNa elutes long before the peptides, allowing less complicated HPLC analysis and product isolation. Furthermore, when thioester formation was attempted on a peptide containing a Ser–Cys motif using MPA at 60 °C, no thioester product was formed. When the same reaction was performed at a higher temperature, a 1 : 1 ratio of thioester to starting material was observed, along with further condensation of the peptide with MPA to form an ester with the hydroxyl of the serine.[12] It has not yet been reinvestigated, but such side reactions would not be expected when using MESNa.

**Scheme 5:**
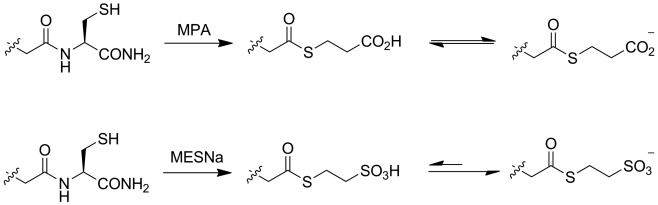
Dissociation of MPA and MESNa thioesters may account for the observed increase in stability of MESNa thioesters towards hydrolysis.

The reason for the increased stability towards hydrolysis could originate from the fact that the sulfonate moiety of MESNa (pKa=−2) would be deprotonated in the pH range of the reaction (pH 2–6) (Scheme 5), whereas MPA would be considerably protonated. The more negatively charged MESNa thioester may stabilize the product against nucleophilic attack by water.

This explanation, in addition to the known acid- or base-promoted degradation of acetylated β-hydroxy thiols,[23] also accounts for the poor stability of thioesters derived from β-mercaptoethanol or dithiothreitol.

**Scheme 6:**
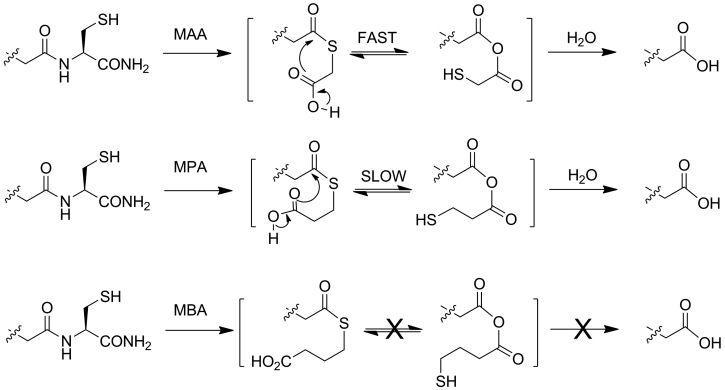
Neighboring group participation of the pendant carboxyl group may catalyze thioester hydrolysis.[24]

Interestingly the homologous thioacids 2-mercaptoacetic acid (MAA, p*K*_a_=3.7), 3-mercaptopropionic acid (MPA, p*K*_a_=4.3), and 4-mercaptobutyric acid (MBA, p*K*_a_ ∼4.7) each displayed very different behavior during thioester formation, despite the similarities in p*K*_a_. With MAA as the thiol additive, only transient thioester formation was observed, followed by rapid hydrolysis. With MPA, hydrolysis only appeared slowly (after 24 h), and when using MBA no hydrolysis was observed. In cases where hydrolysis occurred, it was observed only after significant thioester formation, and so it seemed likely that the thioester product, and not the amide starting material, was undergoing hydrolysis. This was supported by the observation that the starting material appeared completely stable to heating in AcOH (p*K*_a_ 4.76). These observations could be explained by participation of the pendant carboxylic acid, with anhydride formation accelerating hydrolysis (Scheme 6).

Although hydrolysis of thioesters via anhydride intermediates is uncommon, hydrolysis of the anhydride formed by attack of a nearby nucleophilic carboxyl group within an active site of 3-hydroxyisobutyryl-CoA hydrolase on its substrate has been observed.[24a] Consequently, the lower nucleophilicity of the MESNa sulfonate group can also account for its increased stability towards hydrolysis. Despite the favorable features of MBA, it was only poorly water soluble and displayed a tendency to spontaneously form the γ-thiolactone under the reaction conditions.

Regardless of the thiol additive employed, the reaction conditions are generally acidic, and the range at which thioesters are formed is usually from pH 2 to pH 5.8, using 10 % v/v AcOH or 0.1 M Na phosphate buffer to control the pH respectively. However at or above pH 7, the peptide thioester is hydrolyzed, and when N→S acyl transfer was carried out in 1 % v/v TFA (pH 1) in the absence of thiol additives, hydrolysis of the peptide across the Gly–Cys junction was observed. Although sodium phosphate buffer has been employed to maintain approximately pH 5.8, increasing the ionic strength of the buffer (>0.1 M) results in more rapid thioester hydrolysis. In 1.0 M Na phosphate, pH 5.8, the thioester is only a minor component of the reaction mixture and hydrolysis predominates.

The addition of *tris*-(2-carboxyethyl)phosphine (TCEP) to the reaction was found to increase the rate of thioester formation, such that at 50 °C the conversion to thioester was closer to that observed at 60 °C in the absence of TCEP.[20] The beneficial effect of TCEP is tentatively attributed to the prevention of disulfide bond formation by maintaining a reducing environment. However, since TCEP is known to cause the desulfurization of peptides, converting Cys to Ala (usually above pH 7),[25] and to cause the cleavage of peptides and proteins (above pH 7),[26] its use should be monitored carefully. Furthermore, unless solutions of TCEP**⋅**HCl are neutralized before use they can dramatically lower the pH of the reaction.

**Figure 2:**
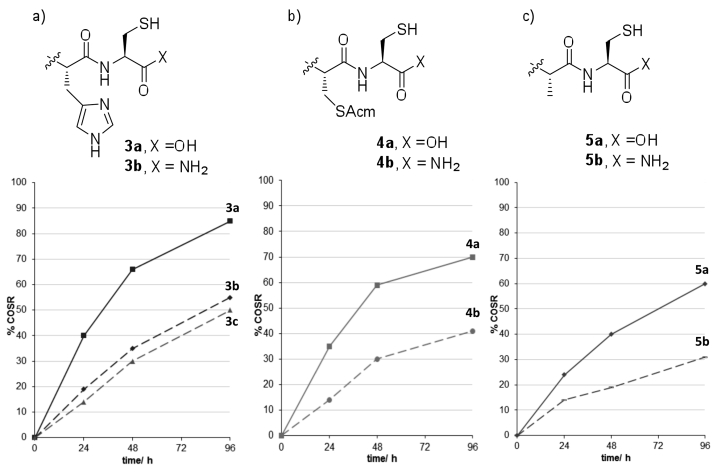
Reaction profiles of Xaa–Cys-terminated peptides prepared as C-terminal carboxylic acids and as C-terminal carboxamides, upon exposure to 10 % w/v MESNa in 10 % v/v AcOH at 60 °C.[17] Note: **3 c** corresponds to a peptide sequence where the His-Cys motif is relocated to the N-terminus of the model peptide, and thioester formation is inferred by observation of the *N-*cysteinyl peptide.

A significant additional improvement is observed in the rate of thioester formation when a precursor contains a C-terminal carboxylic acid, rather than a C-terminal carboxamide (Figure 2).

**Scheme 7:**
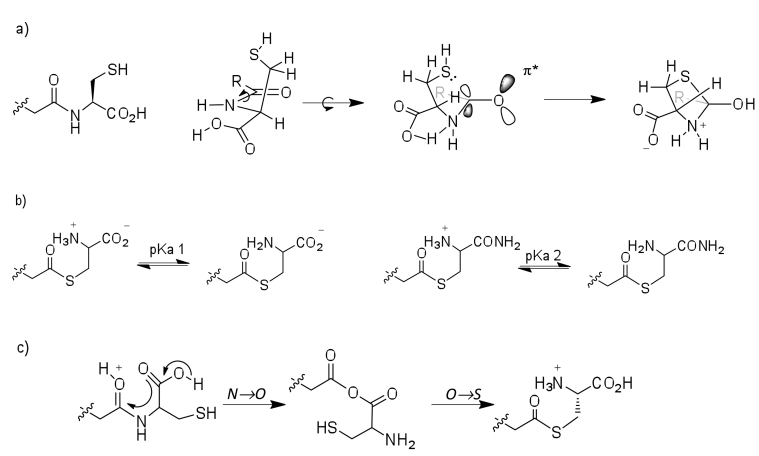
The presence of the carboxyl group appears to accelerate thioester formation, possibly by a) stabilization of the hydroxythiazolidine intermediate, b) increased pKa of the α-amino group (p*K*_a_ 1> p*K*_a_ 2) favoring the *S*-peptide, c) through neighboring group participation.

The reasons for this effect have not been investigated in detail. Intramolecular protonation of the scissile amide nitrogen by the carboxyl group is not unfeasible, although the reaction is already conducted at acidic pH (Scheme 7). Such protonation of the hydroxythiazolidine intermediate by the carboxyl group would result in a zwitterionic intermediate. The *S*-peptide may also exist as a zwitterion and this might positively influence the amide/*S*-peptide equilibrium where p*K*_a_ 1> p*K*_a_ 2 (Scheme 7 b). Alternatively, thioester formation could be facilitated by sequential acyl-transfers involving participation of the carboxyl group (Scheme 7 c),[24b] although this would suggest that all C-terminal amino acids are inherently susceptible to hydrolysis and so is extremely unlikely.

### 2.3. Thioester Formation vs. NCL

**Scheme 8:**
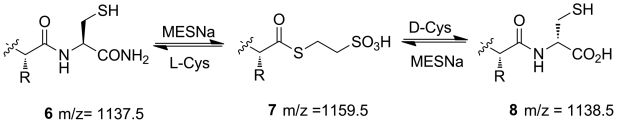
The extent to which NCL competes with thioester formation can be examined by addition of increasing concentrations of d-Cys during thioester formation.[27]

In some instances the reaction shows a certain degree of dependence on the peptide concentration. We were keen to investigate whether NCL can compete with thioester formation, essentially ligating the released cysteine back to the peptide thioester, reforming the starting material.[27] Initially we had considered that this would be unlikely because of the low pH at which thioester formation occurs and the presence of the vast excess (approximately 0.7 M) of MESNa. However, since thioester formation is often observed not to proceed to completion we could not disregard this possibility. To examine the competitive potential of NCL, increasing concentrations of d-cysteine were added into the thioester forming reaction and the amount of epimer formed was measured (Scheme 8). Since d-Cys–OH was added to an l-Cys–NH_2_ terminated peptide (**6**), ligation could be readily observed by both HPLC and mass spectrometry. The d-Cys “epimer” (**8**), formed by NCL, was only observed in significant amounts when the ratio of d-cysteine added to the original peptide was more than 1 : 1. Although in this study the difference in rates of thioester formation between carboxyl- and carboxamide-terminated peptides was not considered, the results clearly indicated that competing NCL occurs under the reaction conditions, but only becomes significant when d-Cys is added in large excess. From these results we concluded that, although thioester formation is reversible, the limited amount of “free” cysteine available (≤1 equiv) under normal reaction conditions precludes significant interference from NCL.

Nagaike and colleagues reported the rate of N→S acyl transfer and thioester exchange to be improved by the use of microwave irradiation.[15] We have found little advantage of microwave irradiation over conventional heating for the formation of peptide thioesters at 60 °C.[20] The reaction can proceed with slower rates at lower temperatures such as 40 °C, which can reduce the hydrolysis of Asp-containing peptides sufficiently to allow for the desired thioester formation on these peptides; however the efficiency of the reaction is significantly compromised at lower temperatures.[12]

The search for optimized reagents and conditions is far from complete. The discovery that small changes in the structure of thiol additive can dramatically affect the outcome, as well as the user friendliness, of the reaction is extremely encouraging. Furthermore, small changes in precursor structure (i.e., carboxyl vs, carboxamide) can also significantly affect the rate of thioester formation. The increased reactivity of carboxyl-terminated substrates is supported by several HPLC, LC-MS, and ^13^C NMR labelling experiments and suggests that additional “engineering” of the cysteine residue or Xaa–Cys motif should be possible.

## 3. “There and Back Again”: Application to Cyclic Peptide Synthesis

Cyclic peptides are highly prized materials, since the conformationally constrained structures can offer enhanced binding to targets, increased stability to proteolysis, and enhanced bioavailability.[28] Although they are of considerable interest as therapeutics, they can be difficult to prepare by synthetic or biological means.[29]

A practical consequence of the lower reactivity of Xaa–Cys–NH_2_ peptides, compared with Xaa–Cys–OH peptides, is that the formation of cyclic amide products from carboxyl-terminated precursors should be favored. Since the cyclic products result in the formation of an internal Xaa–Cys motif at the expense of a terminal Xaa–Cys motif, the preference for cyclic products could be even more pronounced. Previous studies suggested that NCL only competes with thioester formation significantly at high concentrations of added cysteine.[27] However, we considered that the high effective Cys concentration in an intramolecular NCL reaction might render cyclization feasible. Interestingly, the successful synthesis of cyclic peptides of biological origin is most often facilitated by inteins,[30] which utilize the same peptide rearrangement process that we would aim to employ.[31]

Recently we tested this hypothesis with some short, 9–14 residue antimicrobial peptides derived from mouse β-defensin, DefB14 The ability of defensins to kill a broad range of microbes has evolved over millennia, and their precise mechanism of bacterial cell surface disruption remains a matter for debate. We discovered that a small defensin fragment retains much of the antimicrobial activity.[32] and sought to improve its stability through head-to-tail cyclization.

**Scheme 9:**
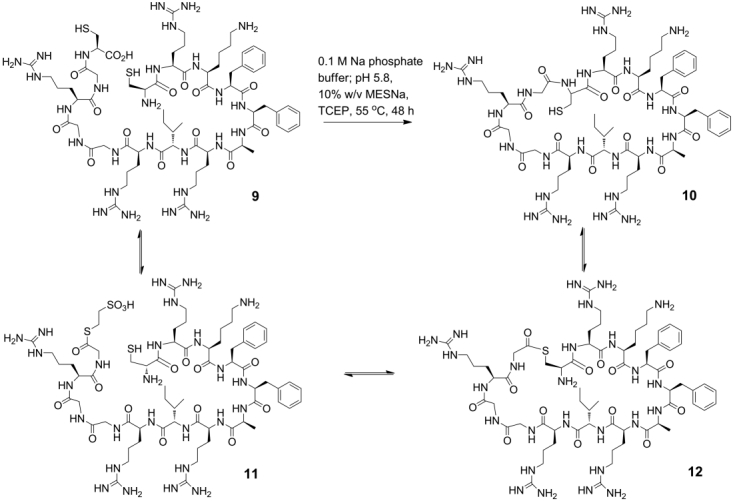
Head to tail cyclization of sequence H–CRKFFARIRGGRGC–OH via N→S acyl transfer followed by NCL.[32b]

In one example (**9**, Scheme 9) we initially examined thioester formation/cyclization at approximately pH 2 (employing 10 % v/v AcOH as solvent) and found that, although the thioester accumulated, only a small degree of cyclization took place. Upon increasing the reaction pH to 5.8 we found that the cyclic peptide **10** emerged from the reaction as the major product (Scheme 9).[32b] We anticipated that raising the pH might reduce the propensity for N→S acyl transfer, but a compensatory increase in the nucleophilicity of the N-terminal cysteine could render NCL more effective. An interesting feature of this reaction is that both *retro*-NCL and NCL appear to proceed in the same reaction vessel yet, under the reaction conditions, are working in concert to produce the cyclic peptide. ^13^C NMR analysis of the reaction mixture does not suggest that the thioester intermediates **11** or **12** accumulate to a significant extent at approximately pH 5.8.

**Figure 3:**
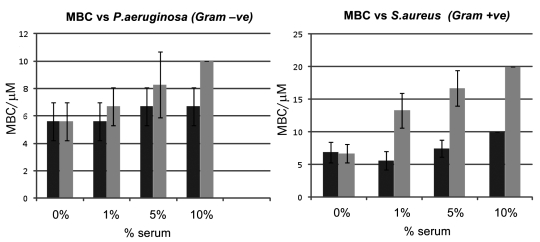
Serum sensitivity assays of linear (light grey) and the *S*-carboxamidomethyl (dark grey) derivative of *ent*-10 against *P. aeruginosa* strain PAO1 and *S. aureus* strain ATCC 25923.

S-carboxamidomethylated derivatives of the cyclic peptides derived from β-defensins retained much of their original activity but, disappointingly, their stability in serum was not significantly enhanced. In contrast we found that the antimicrobial activity of the mirror image cyclic peptide (***ent*****-10**, *P. aeruginosa*, MBC=5.6 μM) remained largely unchanged in 10 % serum (Figure 3). Consequently, we believe this cyclic mirror image peptide serves as an excellent lead “scaffold” from which new antimicrobial agents could be developed.

Although cyclic peptides ultimately emerge from the reaction as major products, it is not obvious why this is the case since the reaction is inherently reversible. It is unlikely that the relative stabilities of Xaa–Cys–NH_2_ and Xaa–Cys–OH alone account for the outcome. Peptides terminating in an Xaa–Cys–NH_2_ motif still undergo cyclization albeit less efficiently, suggesting that the internal nature of the cyclic amide bond within the product is also important. Since water appears to be the best solvent for the reaction, it is possible that the more compact and hydrophobic nature of the cyclic product also inhibits reversion to linear species by excluding water from the reaction site. Furthermore, the conformational constraints imposed by cyclization could present an additional obstacle for the internal Cys, preventing it from attaining an appropriate geometry for N→S acyl transfer.

The results of this study confirmed that several peptides, equipped simply with an N-terminal Cys and an appropriate C-terminal Xaa–Cys motif, are able to undergo head-to-tail cyclization, and that this process is likely applicable to many cyclic peptides of biological or medicinal interest.

## 4

### 4.1. Synthesis of Post-Translationally Modified Peptide Thioesters via N→S Acyl Transfer

Relying on heating peptide samples to 60 °C at mildly acidic pH could certainly raise concern that this method could not be applied to peptides decorated with non-peptide appendages such as carbohydrates. For example, the acetal linkages present in glycopeptides, linking the carbohydrate to the peptide backbone in O-linked glycosylation, or those present in interglycosidic linkages, are well known to be susceptible to acid hydrolysis. In a typical example, benzylidene acetal protected sugars can be cleaved in heated 80 % v/v acetic acid. However, in the case of non-benzylic acetals, catalytic quantities of mineral acids are usually employed to cleave glycosides or acetonides efficiently. Considering that proteins that have undergone post-translational modification (PTM) are of increasing interest for their regulatory and targeting roles in biological systems, it is important that they can be accessed using N→S acyl transfer.

Most PTM occurs on specific amino acid residue side-chains, and two of the most predominant forms of PTM are phosphorylation and glycosylation. Phosphorylation has been shown to be particularly important in the regulation of signal transduction pathways,[5c, 33] while glycosylation plays a key role in diverse biological processes such as protein folding, cell–cell recognition and adhesion, immune defense, and improving the circulatory lifetime of secreted proteins.[34] Phosphorylation is commonly O-linked through Ser, Thr, or Tyr, and complex carbohydrates (glycans) are most commonly N-linked through Asn, or O-linked through Ser and Thr. The site of modification is not under direct genetic control and can be present at one or more residues.[35] For example, erythropoietin (EPO), a glycoprotein hormone, contains three N-glycosylation sites (Asn^24^, Asn^38^, and Asn^83^) and a single O-glycosylation site (Ser^126^).[36] Asparagine residues are glycosylated in response to a required Asn–Xaa–Ser/Thr motif[36c] whereas the sites of O-glycosylation and phosphorylation are dictated more by sequence context.[37] Due to substrate/acceptor competition between the processing enzymes that make up the structure of the glycans, heterogeneously glycosylated structures (glycoforms) are observed.[38] Despite the development of cell culture strategies that can achieve human-like glycosylation, the proteins are isolated as heterogeneous mixtures with respect to glycan structure and glycosylation site occupancy.[39] Phosphoproteins can often be hyperphosphorylated in substoichiometric fashion, which greatly complicates analyses. It is essential to have homogenous forms of these proteins to ensure reliable studies of structure–activity relationships. Consequently, much interest has evolved around the synthesis of homogeneous glycoproteins and phosphoproteins.

Because solid phase peptide synthesis is limited to peptides with up to approximately 50 amino acid residues,[40] NCL is the tool that has been most successful in accessing homogeneous glycoproteins[6b, 41] and phosphoproteins.[5c, 42] Unlike in traditional bioconjugation strategies for protein modification,[43] the native glycopeptide structures can be maintained using NCL or using the thioester method developed by Aimoto.[2a, 44] However, phosphopeptide and glycopeptide thioesters can be more difficult to prepare due to the increased chemical complexity. During peptide synthesis, the appropriate PTM is usually introduced as a suitably protected amino acid building block, and when this is located in the *N*-cysteinyl peptide fragment, synthesis can be extremely straightforward. Often, the real challenge encountered is the synthesis of the PTM containing thioester.

A major factor contributing to the challenge of post-translationally modified peptide thioester synthesis is the fragile nature of these moieties. They are generally incompatible with traditional Boc-based chemistry during which they are repeatedly exposed to TFA and then HF.[45] In an attempt to circumvent this problem, an Fmoc-based strategy is most frequently employed. Most PTM-containing thioesters have been prepared using the acylsulfonamide “safety-catch” linker (Scheme 1). However, manipulations with this linker, including loading and cleavage, can be difficult to monitor in the absence of a double linker strategy,[5f, 46] and “off target” alkylation can occur.[42] Regardless of this, the Fmoc strategy is still generally preferred for glycopeptides and phosphopeptides, and the mildly acidic to neutral conditions employed within N→S acyl transfer methods are highly complementary to the acyl sulfonamide approach.

### 4.2. Phosphopeptide Thioester Synthesis

**Scheme 10:**
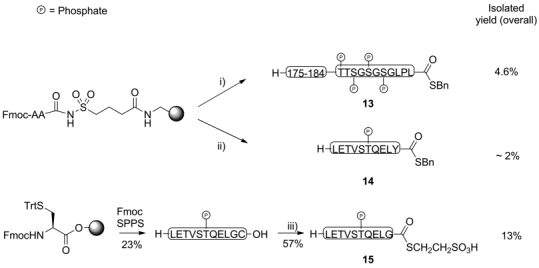
Fmoc phosphopeptide thioester synthesis; *reagents and conditions* i) a) Fmoc SPPS; b) ICH_2_CN, DIPEA; c) BnSH, DIPEA; d) TFA, scavengers. ii) a) Fmoc SPPS; b) ICH_2_CN, DIPEA (twice); c) BnSH, PhSH; d) TFA, scavengers. iii) 10 % w/v MESNa, 0.1 *M* Na phosphate pH 5.8, 55 °C, 72 h.

Muir and co-workers previously used the sulfonamide linker in the semisynthesis of the hyperphosphorylated transmembrane kinase TβR-1.[5c, 42] The phosphorylated GS region of the protein H–TTLKDLIYDM*pTTpSGpSGpSGLPL–SBn (residues 175–195, **13**) were synthesized using an optimized procedure in a yield of 4.6 % (Scheme 10). In order to be successful, the methionine residue was substituted for norleucine to preclude a previously observed side reaction between methionine and iodoacetonitrile during resin activation. The thioester was subsequently ligated to the remainder of the TβR-1 cytoplasmic domain, obtained by recombinant methods.

Macmillan and co-workers also attempted to use the safety-catch linker but experienced low yields of ∼2 % in the synthesis of a short thioester (H–LETVS*p*TQELY–SBn, **14**).[47] Upon use of N→S acyl shift, the thioester **15** (H–LETVS*p*TQELG–SCH_2_CH_2_SO_3_H), which included a Y27G substitution to facilitate thioester formation, was synthesized in an unoptimized overall yield of 13 %.[48] The phosphopeptide thioester was ligated to a recombinant Chk2 kinase ForkHead Associated (FHA) domain to produce a stoichiometrically phosphorylated protein that formed a characteristic phosphate-dependant homodimer.

### 4.3. Glycopeptide Thioester Synthesis

**Scheme 11:**
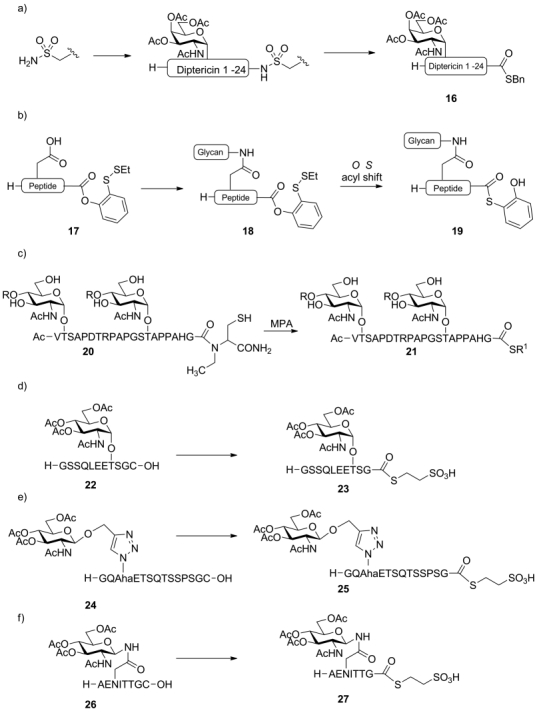
Fmoc-based approaches to glycopeptide thioester synthesis: a) using the sulfonamide safety-catch linker;[5b] b) phenolic ester;[16a] c) *N*-alkyl cysteine, R=β-d-Gal, R^1^=CH_2_CH_2_CO_2_H;[51] d–f) Cys terminated peptides.[27, 48]

Using the sulfonamide linker to produce a thioester and NCL, Shin et al.[5b] were able to synthesize an 82-residue antibacterial glycopeptide containing two O-linked GalNAc residues at Thr^10^ and Thr^54^. The thioester fragment **16** (diptericin residues 1–24) was isolated in 21 % yield (Scheme 11). A similar approach also allowed Macmillan and Bertozzi to prepare homogeneous semisynthetic glycoforms of mouse glycosylation-dependant cell adhesion molecule 1 (GlyCAM-1) containing O-linked sugars.[13]

Later, Unverzagt and co-workers were able to synthesize a peptide thioester containing an N-linked biantennary heptasaccharide to make a fragment of RNase B. The safety-catch linker was activated with trimethysilyldiazomethane and the glycopeptide thioester was cleaved from the resin with ethyl 3-mercaptopropionate and sodium thiophenolate. The side-chain protecting groups were then removed under acidic conditions with TFA and purification of the thioester yielded 46 %.[5f] However, since TFA cleavage conditions have been found to be incompatible with certain glycosidic linkages (for example, fucosidic linkages in EPO), Danishefsky et al.[49] explored the possibility of incorporating a glycan after peptide synthesis via Lansbury aspartylation.[50] An unsymmetrical aryl–alkyl disulfide was coupled to a peptide to form **17**, which was subsequently glycosylated with a chitobiose-derived glycosylamine to give **18**. After reduction under ligation conditions, it was proposed that **18** underwent intramolecular O→S acyl shift to generate thioester **19** in situ, which was then able to participate in NCL.

Ozawa et al. were able to use an N→S acyl shift to generate glycopeptide thioesters.[51] After installation of *N*-alkyl cysteine (NAC) on CLEAR amide resin, the rest of the peptide chain was elongated using standard Fmoc protocols. The carbohydrate moieties were introduced using the appropriate glycoaminoacid building block. After complete assembly, the product **20** was cleaved from the solid support and dissolved in 5 % MPA solution, facilitating conversion to the thioester **21** in 2 days. This proceeded without significant decomposition of carbohydrate appendages, with an overall yield of 20 % (calculated from initial resin loading).

Masania et al. were also able to apply N→S acyl transfer in the context of glycopeptides. Glycosylation-dependant cell adhesion molecule 1 (GlyCAM-1) residues 75–82 H–GSSQLEE**T**SGC–OH (**22**) were assembled as a model O-linked glycopeptide incorporating Fmoc–GalNAc(OAc)_3_-Thr–OH at position 82.[48] An analogue of cell surface glycopeptide CD52 (**24**) was also prepared, substituting Asn^3^ with azidohomoalanine (Aha). This enabled the attachment of *N*-acetyl glucosamine through an unnatural triazole linkage formed through Cu^I^-mediated cycloaddition.[52] After being cleaved from the solid support, both glycopeptides were subjected to thioesterification conditions (10 % w/v MESNa, 0.1 M Na phosphate; pH 5.8, 55 °C for 72 h) to generate thioesters **23** (10 %) and **25** (18 %), respectively.

More recently we investigated N→S acyl shift in the preparation of N*-*linked glycopeptides.[27] EPO residues 22–28 H–AEN(GlcNAc(OAc)_3_)ITTGC (**26**) were assembled on NovaSynTGT resin and subjected to thioesterification (10 % w/v MESNa, 0.5 % w/v TCEP, 0.1 M Na Phosphate; pH 5.8, 55 °C for 48 h) to give thioester **27** with a good overall yield of 21 %. Conducting the reaction at pH 5.8 was important, since at lower pH the acetyl esters were cleaved, giving rise to a complex mixture of partially deacetylated compounds. It would have been extremely convenient if quantitative deacetylation had accompanied thioester formation, but deacetylation proceeded slowly (at pH 2) and longer reaction times resulted in increased thioester hydrolysis. Although only a single monosaccharide was introduced, glycopeptide/protein remodelling strategies can elaborate these simple glycopeptides into more complex structures.[53]

Overall, use of N→S acyl transfer in the formation of post-translationally modified peptide thioesters has demonstrated versatility in application to phosphoproteins, glycoproteins, and their analogues. We have also shown the tolerance of a variety of non-peptidic linkages to this reaction. Moreover the efficiency of this process is reflected in good yields without the requirement for specialized resins, linkers, and unnatural building blocks.

## 5. Summary and Outlook

N→S acyl transfer is clearly emerging as a useful route to peptide thioesters, which are important intermediates in protein synthesis. It is especially interesting that native peptides have the ability to rearrange into thioesters, since this suggests that proteins have always had, at least in theory, an inherent ability to make themselves through N→S acyl transfer and NCL. The required information for the production of the key components for NCL, which can be challenging to produce with modern synthetic methods, is already installed in an Xaa–Cys motif.

The biological relevance or consequence of this process is yet to be examined in detail. One could take the view that cysteine is an amino acid whose rogue properties require that it is “tied up” in disulfide bonds or coordinated to metals if it cannot be deleted entirely. It would be expected that hyperthermophiles would evolve to remove all but the most essential Cys residues. Indeed, hyperthermophiles have only a fraction of the cysteine compared with mesophiles. An alternative view is that, as life thrived in a more temperate environment, new opportunities have emerged for this uniquely reactive amino acid.

Additionally, proteins have been exposed as being inherently hydrolytically labile across Xaa–Cys sites in a manner that was not widely appreciated in the past. This may find application in proteomics as well as in the thioester syntheses that we have explored thus far.

Major challenges to address in thioester formation from native peptide sequences are the requirements for elevated reaction temperatures and acidic pH, especially when several “device”-driven routes produce thioesters at ambient temperature and neutral pH. An advantage of using native sequences is that they can be genetically encoded and therefore applied to samples of biological origin, but several obstacles (described in Section 2.1) still need to be addressed for this to become generally applicable. Further engineering of N→S acyl transfer devices is likely to continue apace, and both improved processes and additives that can influence the acyl transfer equilibrium will emerge.

Unlike when using inteins in bring about thioester synthesis, most of the processes discussed above cannot currently be applied in cell culture. But it is not unimaginable that an optimized, caged acyl transfer facilitating device could be genetically encoded such that C-terminal protein thioesters or thioester precursors could be produced in the absence of inteins.[54]
